# Actinomycetoma Due to Nocardia brasiliensis: Recognition in Primary Care

**DOI:** 10.7759/cureus.103509

**Published:** 2026-02-12

**Authors:** Jesús Iván Martínez-Ortega, Frida Itzel Rosas-Lezama, Alejandra Nicole Macias Quiroga

**Affiliations:** 1 Histology, Autonomous University of Nuevo Leon, Monterrey, MEX; 2 Dermatology, Dermatology Institute of Jalisco, Zapopan, MEX; 3 Dermatology Service, General Zone Hospital 11, Mexican Institute of Social Security, Piedras Negras, MEX; 4 Department of Internal Medicine, General Zone Hospital, Mexican Institute of Social Security, Merida, MEX; 5 Dermatology Department, Hospital Obrero No 1, La Paz, BOL

**Keywords:** actinomycetoma, chronic subcutaneous infection, diagnostic delay, family medicine, latin america, neglected tropical diseases, nocardia brasiliensis, primary care, rural health, tropical medicine

## Abstract

Mycetoma is a chronic, progressively destructive subcutaneous infection caused by aerobic filamentous bacteria (actinomycetoma) or true fungi (eumycetoma). It represents one of the most neglected tropical diseases, disproportionately affecting vulnerable populations in resource-limited rural settings. In Latin America, actinomycetoma predominates, most commonly caused by *Nocardia brasiliensis*. Delayed diagnosis is frequent due to its indolent course and limited awareness at the primary care level, often resulting in severe deformity, disability, or amputation.

We report the case of a 43-year-old agricultural worker from rural Bolivia who presented with a two-year history of progressive, painless swelling of the right foot and ankle, associated with multiple draining sinus tracts extruding whitish grains. Based on the classic clinical triad and epidemiological context, mycetoma was suspected in the primary care setting. Direct microscopic examination supported actinomycetoma, and empirical antibiotic therapy was initiated. Culture later confirmed *Nocardia brasiliensis*. Histopathology demonstrated a basophilic grain surrounded by suppurative and granulomatous inflammation.

This report underscores the critical role of primary care physicians in the early clinical recognition of mycetoma using basic diagnostic tools, particularly in endemic and underserved regions. Prompt identification and timely referral of actinomycetoma can substantially reduce morbidity, prevent irreversible tissue damage, and improve functional outcomes.

## Introduction

Mycetoma, historically known as “Madura foot,” is a chronic subcutaneous infection caused by either aerobic filamentous bacteria (actinomycetoma) or true fungi (eumycetoma). Since 2016, it has been classified as a neglected tropical disease, reflecting its close association with poverty, limited access to healthcare, and occupational exposure in rural settings [1-3]. Clinically, mycetoma is characterized by a gradually progressive granulomatous inflammatory process that may extend from the skin to deeper soft tissues and bone, resulting in severe deformity, functional impairment, and permanent disability when diagnosis and treatment are not initiated promptly [1-4].

Mycetoma exhibits an uneven and heterogeneous global distribution. It is endemic in tropical and subtropical regions within the so-called “mycetoma belt,” which extends approximately between latitudes 15° South and 30° North. This belt includes extensive regions of Africa, Latin America, and Asia, where climatic conditions, agricultural practices, and limited access to healthcare interact to promote the persistence of the disease. However, cases have been reported worldwide, including in non-endemic regions, largely due to migration and increased global mobility. To date, mycetoma has been documented in more than 100 countries, underscoring that it should not be regarded as a disease confined to specific geographic areas [3,5].

In Latin America, actinomycetoma is the most common clinical presentation, with *Nocardia brasiliensis* being the most frequently identified causative organism [1,2,5]. The causative organisms are environmental, residing in soil and plant material, and infection is believed to occur primarily through traumatic implantation, often following minor or unnoticed injuries sustained during agricultural activities [1,3,5]. Occupational exposure among farmers and rural workers remains a major epidemiological determinant in endemic regions. Although many patients do not recall a specific traumatic event, the prolonged and poorly defined incubation period likely contributes to the absence of a clearly identifiable history of trauma [1,2].

The classic clinical triad of painless subcutaneous swelling, multiple sinus tracts, and extrusion of grains is highly suggestive of mycetoma and remains a key diagnostic clue. In early stages, mycetoma is frequently misdiagnosed as a bacterial abscess, chronic osteomyelitis, sporotrichosis, or other subcutaneous infections, contributing to delayed diagnosis in primary care. Distinguishing actinomycetoma from eumycetoma is clinically important because management differs substantially: actinomycetoma typically responds to prolonged antibacterial therapy, whereas eumycetoma often requires antifungal treatment and sometimes surgical intervention. Recognition of this triad is particularly valuable in primary care and resource-limited settings, where access to advanced diagnostic tools may be restricted, and early clinical suspicion can guide timely management and referral. Early identification at the primary care level is crucial to reduce diagnostic delay and prevent progression to advanced disease [1-4]. This case report aims to highlight the importance of early clinical recognition of mycetoma in primary care, the need for timely referral in rural settings, and the consequences of delayed diagnosis in resource-limited environments.

## Case presentation

A 43-year-old man from a rural area of Bolivia, employed as an agricultural worker, presented to a primary care clinic with a progressively enlarging mass involving the right foot and ankle, with an estimated duration of approximately two years. He had no relevant past medical history, denied diabetes mellitus, immunosuppression, HIV infection, or chronic medication use, and reported no history of smoking or alcohol abuse. The lesion had initially appeared as a small, painless subcutaneous nodule that had slowly enlarged, evolving into a diffuse deformity with multiple coalescent nodules and draining sinus tracts. These sinuses intermittently discharged whitish granular material. The patient denied fever, significant pain, pruritus, or systemic symptoms such as weight loss or chronic cough. He did not recall a specific traumatic event but reported frequent barefoot or poorly protected exposure to soil and vegetation during agricultural work.

**Figure 1:**
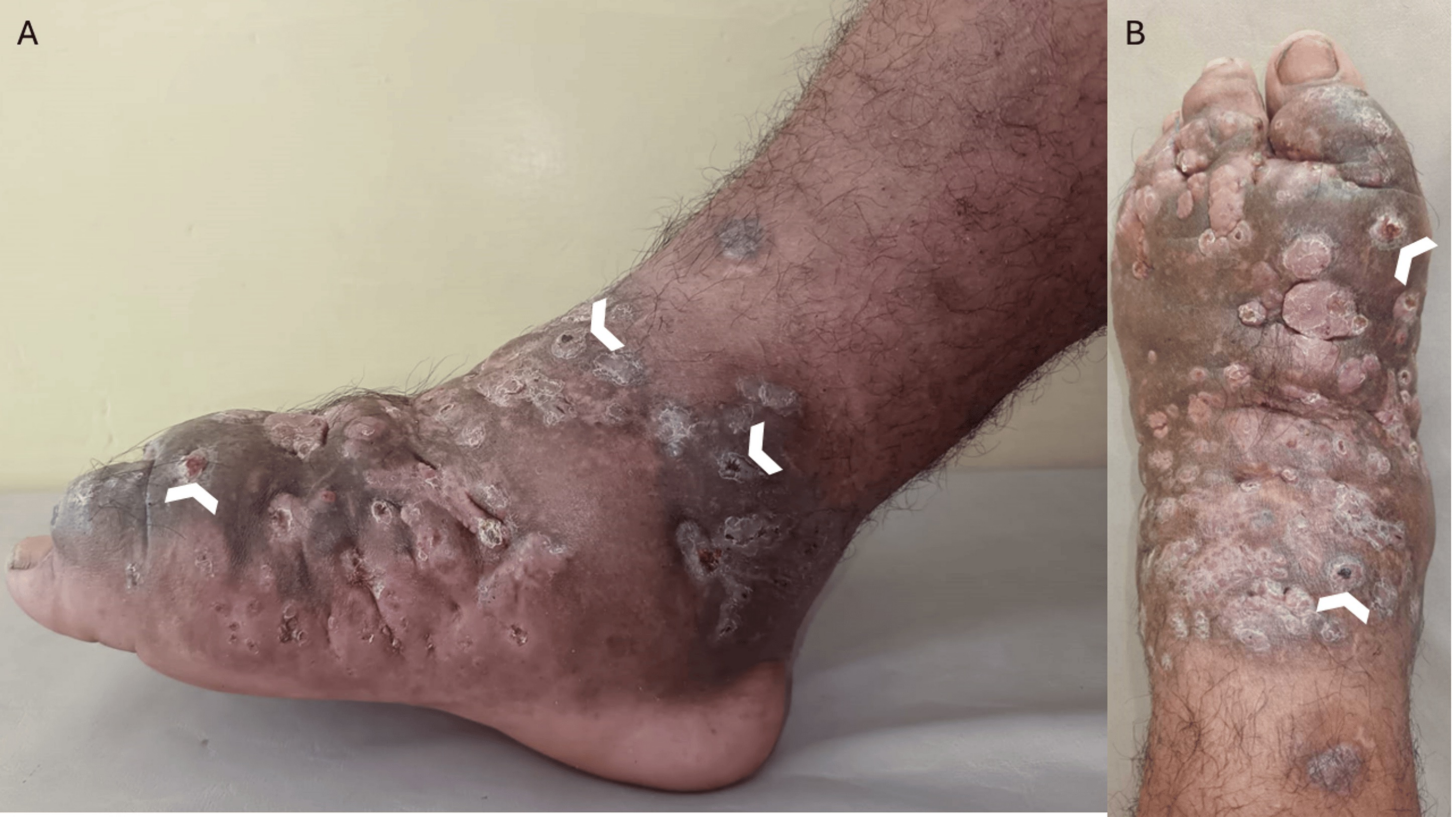
Clinical presentation of actinomycetoma of the right foot Anterior view of the right foot showing diffuse swelling with multiple crateriform sinus openings (white arrowheads). B: Lateral view demonstrating painless tumefaction with multiple draining sinus tracts (white arrowheads), consistent with the classic clinical triad of mycetoma

Physical examination revealed diffuse swelling and deformity of the right foot, with indurated skin and multiple crateriform sinus openings consistent with draining fistulas. No regional lymphadenopathy or signs of acute infection were observed (Figure 1).

Given the chronic course, epidemiological context, and the presence of the classic clinical triad-painless swelling, sinus tract formation, and grain extrusion-mycetoma was suspected. Direct microscopic examination of the grains using 10% potassium hydroxide demonstrated granular structures compatible with actinomycetes. Samples were obtained and sent to an urban reference microbiology laboratory for culture and histopathological evaluation.

Baseline laboratory tests, including renal function, were within normal limits. After microbiological confirmation, empirical antibiotic therapy was initiated in the primary care setting with oral trimethoprim-sulfamethoxazole (160/800 mg every 12 hours) combined with intramuscular amikacin (15 mg/kg/day), while referral to dermatology and infectious diseases services at a higher-level center was arranged for further evaluation and management. Identification of *Nocardia brasiliensis* was established using conventional microbiological methods based on grain morphology, culture characteristics, and microscopic features, as advanced identification techniques such as MALDI-TOF or molecular testing were not available. Renal function was monitored periodically during treatment (every two to four weeks), and the patient was clinically assessed at each visit for auditory or vestibular symptoms suggestive of aminoglycoside toxicity.

**Figure 2:**
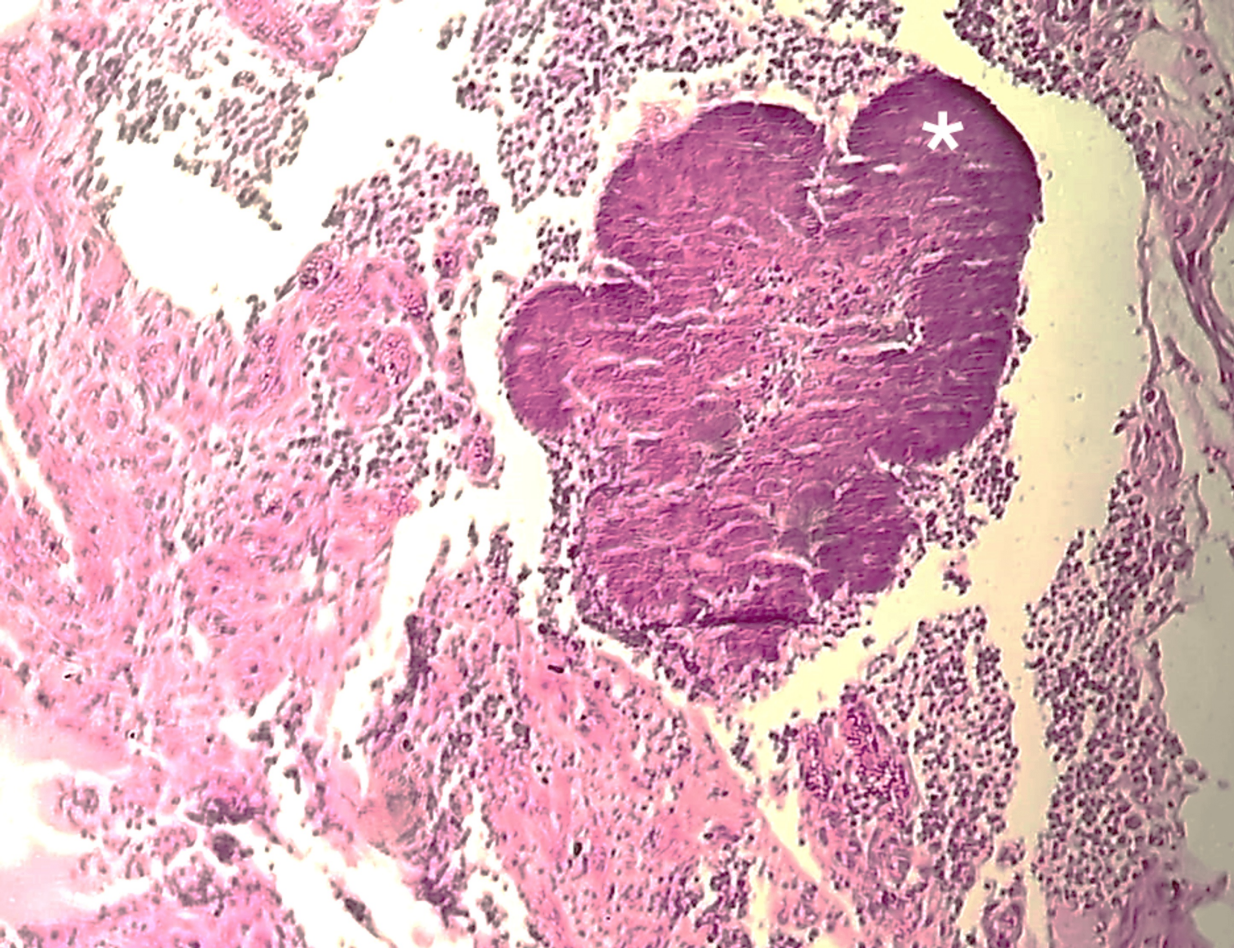
Histopathological findings (hematoxylin-eosin stain at 4x magnification) A compact basophilic actinomycetoma grain is observed in the deep dermis (white asterisk), surrounded by intense suppurative and granulomatous inflammation with neutrophilic abscesses and multinucleated giant cells

Histopathological examination with hematoxylin and eosin staining showed a compact basophilic grain located in the deep dermis, surrounded by a suppurative granulomatous inflammatory reaction with neutrophilic abscesses and multinucleated giant cells (Figure 2).

At one-month follow-up, the patient showed partial clinical improvement, with an estimated reduction of approximately 10% in lesion size. Draining sinus tracts had become inactive, and no new discharge was observed. The patient did not report any systemic symptoms or adverse effects related to treatment. Unfortunately, further follow-up was not achieved, most likely due to geographic and socioeconomic challenges frequently encountered in rural settings.

## Discussion

Mycetoma represents a significant diagnostic challenge in primary care due to its indolent, painless, and slowly progressive course, which frequently leads to prolonged diagnostic delays. Initial clinical assessment should integrate lesion chronology, epidemiological background, and history of repeated microtrauma or soil exposure, enabling early differentiation from aggressive neoplastic processes or systemic infections [1-4,6]. Recent global analyses have highlighted that diagnostic delays of several years remain common in endemic regions, contributing substantially to advanced disease, bone involvement, and irreversible functional impairment [5].

Among chronic implantation infections, the differential diagnosis includes cutaneous tuberculosis, botryomycosis, chromoblastomycosis, and sporotrichosis, particularly in rural and agricultural settings in Latin America, where traumatic inoculation by plant material is common [1,6-9]. However, the presence of the classic clinical triad - painless swelling, multiple sinus tracts, and extrusion of grains - is highly suggestive of mycetoma and helps distinguish it from these entities, in which granular discharge is not typically observed [1,4,6,10]. Recognition of this triad is especially relevant in primary care and rural settings, where access to imaging, histopathology, or molecular diagnostics may be limited.

Macroscopic identification of grains and their analysis by direct microscopy represent accessible diagnostic tools in the primary care setting, aiding in differentiation between actinomycetoma and eumycetoma. From an etiological standpoint, actinomycetoma is characterized by relatively faster progression, abundant purulent discharge, and small, pale, friable grains composed of fine bacterial filaments. In contrast, eumycetoma generally follows a more indolent course, characterized by larger, dark, firm grains, less purulent drainage, and limited responsiveness to antibiotics, often necessitating prolonged antifungal therapy and, in many cases, extensive surgical management [1,2]. This distinction is clinically significant, as delayed or inaccurate differentiation may lead to inappropriate treatment and increased morbidity.

Actinomycetoma generally responds favorably to systemic antibiotics, with trimethoprim-sulfamethoxazole (TMP-SMX) as the cornerstone of treatment. Combination regimens are frequently employed to enhance efficacy and reduce the risk of resistance, and early initiation of therapy is associated with improved outcomes and reduced tissue destruction. In endemic areas such as Latin America, where actinomycetoma clearly predominates, starting empirical antibiotic treatment in cases of strong clinical suspicion is a reasonable approach while awaiting etiological confirmation, as it supports timely referral and helps prevent progressive, destructive disease (Table 1) [1,2].

**Table 1:** Stepwise diagnostic approach to suspected mycetoma in primary care This algorithm reflects a pragmatic approach based on clinical evaluation and basic microscopy, including direct grain examination with potassium hydroxide (KOH) when available. Advanced imaging, culture, and specialized microbiological identification may not be accessible in low-resource settings and are typically performed after referral to higher-level centers. Empirical antibiotic therapy may be initiated in primary care when actinomycetoma is clinically suspected and basic safety monitoring is feasible. Suspicion of deep or bone involvement does not preclude treatment initiation but warrants prompt referral for imaging, multidisciplinary evaluation, and long-term management. Referral is recommended in cases of extensive disease, suspected deep extension, diagnostic uncertainty, risk of drug toxicity, or inability to ensure appropriate clinical and laboratory monitoring.

Step	Clinical question	Key findings	Suggested action
1	Is the lesion chronic and progressive?	Evolution > months, painless swelling	Consider chronic implantation infection
2	Is there an epidemiological risk?	Rural origin, agricultural work, barefoot exposure	Raise suspicion for mycetoma
3	Are sinus tracts present?	Multiple draining openings	Examine for grain extrusion
4	What is the grain appearance?	Small, pale, or whitish grains	Suspect actinomycetoma
5	Can basic microscopy be performed?	KOH/direct exam available	Differentiate actino vs. eumycetoma
6	Is deep extension suspected?	Deformity, long evolution, reduced mobility	If imaging is unavailable, refer for extension studies
7	Can safe treatment monitoring be ensured?	Renal function tests and aminoglycoside monitoring available	If yes - start empirical therapy; if no - urgent referral
8	Confirmatory studies	Culture and histopathology	Refer to dermatology/infectious diseases

Nevertheless, treatment often requires prolonged courses of potentially toxic antibiotics, and adverse effects, the need for regular monitoring, and limited access to healthcare services may compromise adherence, particularly in resource-limited rural settings. These challenges further underscore the importance of early clinical recognition and timely management to minimize disease extent and therapeutic burden [10,11].

Despite Bolivia being geographically located within the so-called “mycetoma belt” and sharing ecological, occupational, and socioeconomic characteristics with neighboring endemic countries such as Brazil, Venezuela, and Mexico, published epidemiological data on mycetoma from Bolivia are strikingly scarce. This absence likely reflects underdiagnosis and underreporting rather than true disease absence [5]. Our case, therefore, highlights the need for increased awareness among primary care physicians and supports the rationale for future epidemiological studies and strengthened surveillance systems to better define the burden of mycetoma in Bolivia and similar settings.

Preventive strategies, including health education, the use of protective footwear in exposed populations, and the strengthening of referral networks - potentially supported by telemedicine in remote rural settings - are essential for reducing disease-associated morbidity. The consistently reported diagnostic delays underscore the importance of maintaining a high index of suspicion in primary care when evaluating slowly progressive chronic cutaneous lesions [1,11].

This report has several limitations inherent to resource-limited rural settings. Advanced imaging studies, such as CT or MRI, were not available locally, preventing assessment of the exact depth of involvement and possible bone extension at the time of initial evaluation. Microbiological identification relied on conventional phenotypic methods, as MALDI-TOF and molecular diagnostics were not accessible. Additionally, long-term follow-up could not be completed due to geographic and socioeconomic barriers. Nevertheless, these constraints reflect real-world conditions in many endemic regions and underscore the importance of strengthening primary care recognition, safe initial management, and timely referral pathways for suspected mycetoma.

## Conclusions

Mycetoma should be suspected in primary care when evaluating chronic, painless, and progressive cutaneous lesions in patients with rural exposure or risk factors for traumatic implantation. In this case, recognition of the classic clinical triad enabled early clinical suspicion, basic diagnostic orientation, initial antimicrobial therapy, and referral for specialized evaluation. Although short-term clinical improvement was observed, long-term outcome and therapeutic response could not be assessed due to loss to follow-up. This report highlights the critical role of primary care physicians in early recognition, initial etiological orientation, and timely referral of suspected mycetoma, particularly in endemic and resource-limited settings.
